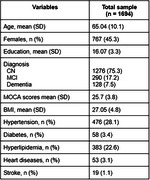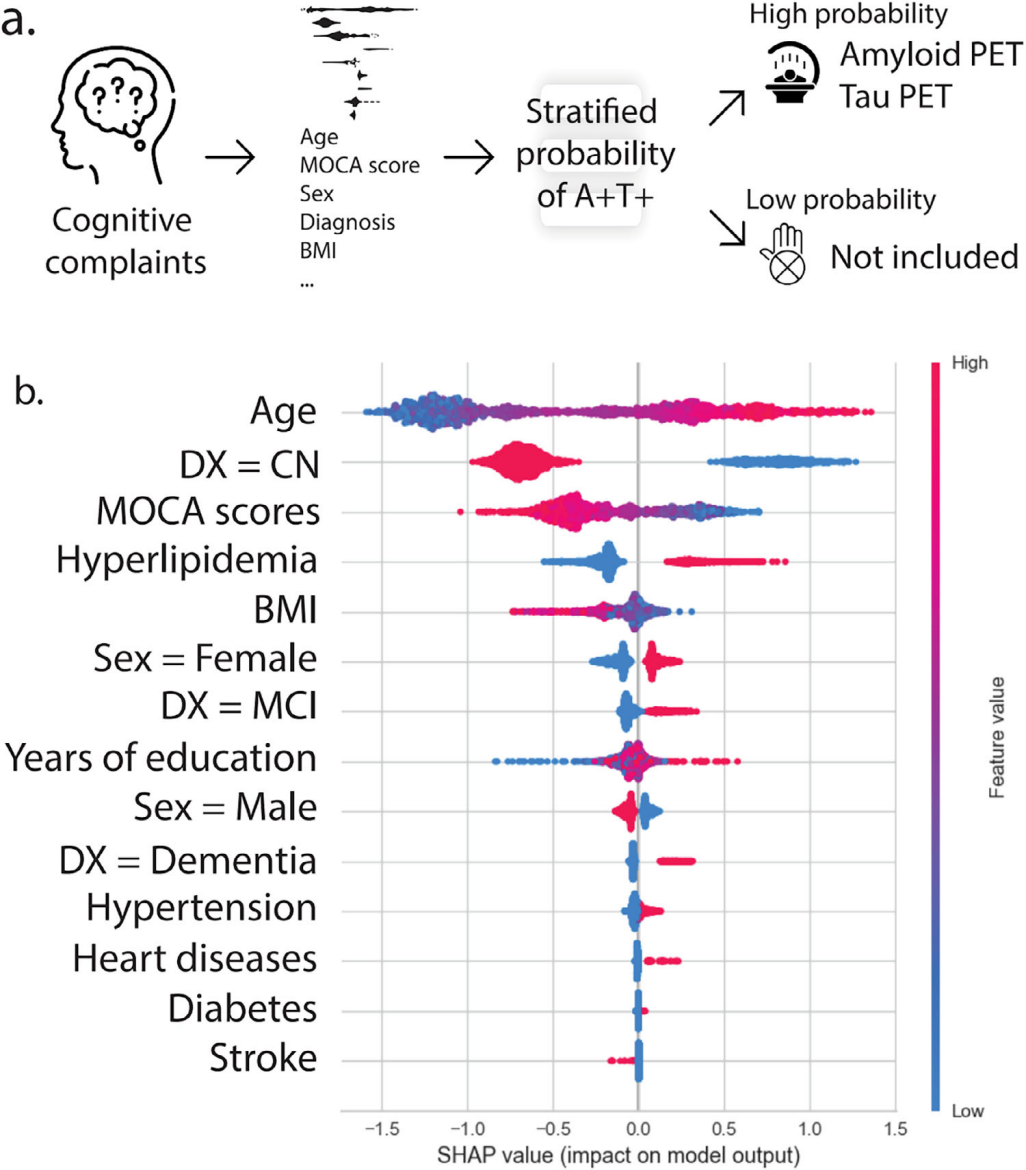# A machine learning approach to predict amyloid and tau positivity using clinical features

**DOI:** 10.1002/alz.091844

**Published:** 2025-01-09

**Authors:** Daniel Arnold, Wyllians Vendramini Borelli, Luiza Santos Machado, Nesrine Rahmouni, Joseph Therriault, Stijn Servaes, Jenna Stevenson, Arthur C. Macedo, Artur Francisco Schumacher‐Schuh, Christian Mattjie, Rodrigo C. Barros, Marco Antônio de Bastiani, Firoza Z Lussier, Mira Chamoun, Gleb Bezgin, Andrea L. Benedet, Tharick Pascoal, Pedro Rosa‐Neto, Eduardo R. Zimmer, Alzheimer's Disease Neuroimaging Initiative (ADNI)

**Affiliations:** ^1^ Federal University of Rio Grande do Sul, Porto Alegre, Rio Grande do Sul Brazil; ^2^ McGill University, Montreal, QC Canada; ^3^ Translational Neuroimaging Laboratory, The McGill University Research Centre for Studies in Aging, Montréal, QC Canada; ^4^ HCPA, Porto Alegre, Rio Grande do Sul Brazil; ^5^ PUCRS, Porto Alegre, Rio Grande do Sul Brazil; ^6^ University of Pittsburgh, Pittsburgh, PA USA; ^7^ Department of Psychiatry and Neurochemistry, Institute of Neuroscience and Physiology, The Sahlgrenska Academy, University of Gothenburg, Mölndal, Gothenburg Sweden; ^8^ Federal University of Rio Grande do Sul (UFRGS), Porto Alegre, RS Brazil; ^9^ Laboratory of Neuro Imaging (LONI), University of Southern California, Los Angeles, CA USA

## Abstract

**Background:**

Screen failure due to amyloid negativity is yet a problem in clinical trials for anti‐amyloid drugs. In this context, clinical characteristics of patients presenting with cognitive decline may decrease the screen failure ratio by increasing the odds of selecting individuals with brain amyloid pathology. Herein, we aimed at estimating amyloid and tau positivity in individuals using clinical variables in a machine learning model of prediction.

**Method:**

We selected 1694 participants with amyloid and tau status from ADNI, TRIAD, PPMI databases. Commonly shared clinical features selected between datasets were age, total MOCA scores, clinical diagnosis, sex, education, BMI, heart disease, stroke, hyperlipidemia and diabetes. Amyloid positivity was defined by amyloid‐PET (PIB‐PET, FBB‐PET or AZD4694‐PET) or CSF AB42 and Tau positivity defined by Tau‐PET (MK6240‐PET or AV1451‐PET) or CSF p‐tau181. The dataset was split into training (49%), validation (21%), and testing datasets (30%). A XGBoost model was tuned, and then used to predict the tau status outcome in the testing dataset.

**Result:**

A total of 927 men and 767 women were included with mean MOCA 25.7 ± 3.8 scores (Table). The population consisted of 1276 CN, 290 MCI and 128 dementia individuals (1357 A‐T‐ and 337 A+T+). The receiver‐operator characteristic (ROC) analysis showed that the area under the curve (AUC) was 0.86 for discriminating A‐T‐ vs. A⁺T⁺, with 0.82 as sensitivity, specificity as 0.90 and accuracy as 0.88. The top 3 most impactful features were found to be age, diagnosis and MOCA score through a SHAP value analysis (Figure).

**Conclusion:**

Predicting amyloid and tau positivity with clinically collectible variables may improve selection of individuals for anti‐amyloid trials. A two‐step workflow using this approach may significantly reduce the costs of AD trials. The following studies may incorporate an individualized calculator using clinical variables to estimate amyloid and tau positivity.